# Supplementary figures and images for: Comparison of 3D laser-based photonic scans and manual anthropometric measurements of body size and shape in a validation study of 123 young Swiss men
Source: PeerJ. 2017 Feb 9;5:e2980. doi: 10.7717/peerj.2980 (PMC5345820; doi:10.7717/peerj.2980)

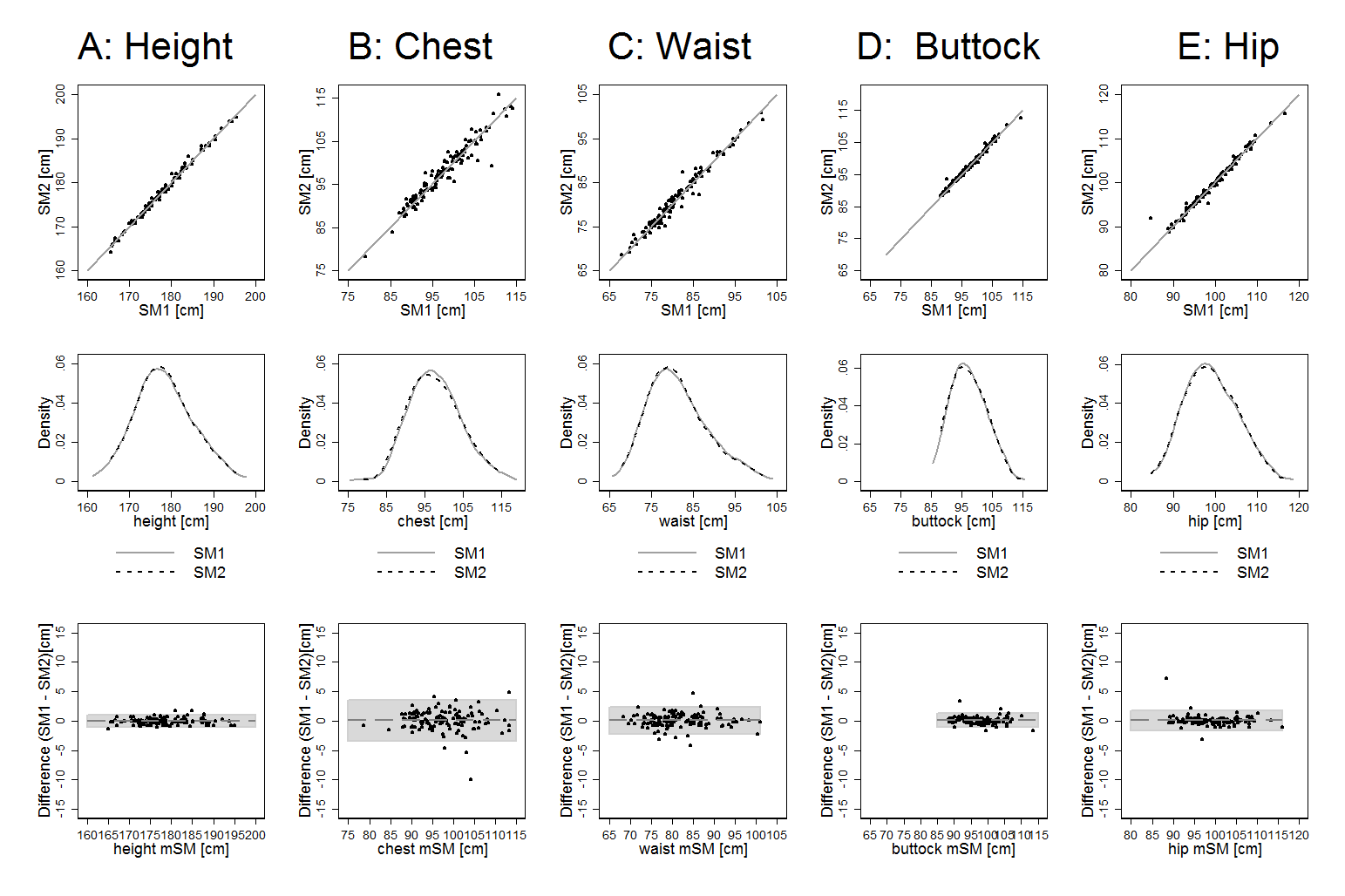

Supplement: Figure S1 — Agreement between the repeated scan measurements (SM1 and SM2) by scatterplots (top row), kernel density plots (middle row, bandwidth = 3), and BA plots (bottom row). The detailed results are displayed in Table 3. [file peerj-05-2980-s003.png]

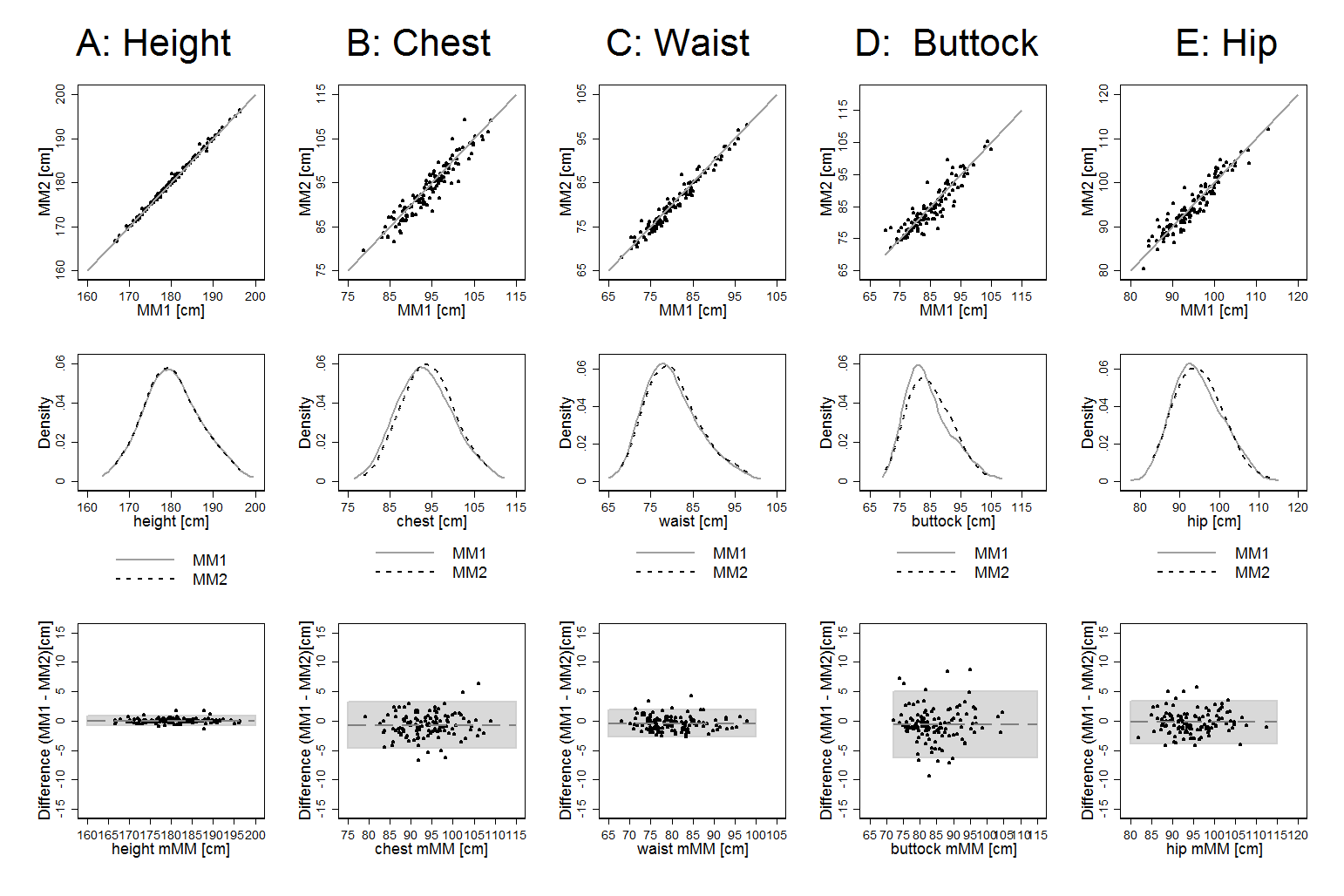

Supplement: Figure S2 [file peerj-05-2980-s004.png]
